# Phase Behavior and Thermodynamic Model Parameters in Simulations of Extractive Distillation for Azeotrope Separation

**DOI:** 10.1038/s41598-017-09088-2

**Published:** 2017-08-25

**Authors:** Min Li, Xicai Xu, Xin Li, Kang Ma, Bin Qin, Zhaoyou Zhu, Yinglong Wang

**Affiliations:** College of Chemical Engineering, Qingdao University of Science and Technology, Qingdao, 266042 China

## Abstract

Extractive distillation (ED) processes for separating ternary mixtures of benzene-cyclohexane-toluene with dimethyl formamide (DMF) and N-methyl-2-pyrrolidone (NMP) were studied using Aspen Plus and PRO/II simulators. The Aspen Plus built-in binary interaction parameters for the toluene-DMF, benzene-NMP and cyclohexane-NMP systems resulted in inaccurate phase behavior calculations. The vapor-liquid equilibrium (VLE) for the three binary systems was regressed to illustrate the importance of using accurate model parameters. The obtained binary interaction parameters described the phase behavior more accurately compared with the built-in binary interaction parameters in Aspen Plus. In this study, the effects of the regressed and built-in binary interaction parameters on the ED process design are presented. The total annual cost (TAC) was calculated to further illustrate the importance of the regressed binary interaction parameters. The results show that phase behavior and thermodynamic model parameters should receive more attention during the research and development of ED processes.

## Introduction

Distillation^1^, which is based on the relative volatility differences between components in a mixture, is one of the most important separation technologies. However, it is difficult to separate mixtures efficiently using conventional distillation when they have similar boiling points or form azeotropes. To separate azeotropes, several advanced distillation technologies have been studied, such as pressure-swing distillation^2–7^, azeotropic distillation^8, 9^ and extractive distillation (ED)^10–15^. ED is achieved by adding an appropriate solvent with a higher boiling point to enhance the relative volatility of the components.

ED is one of the most economical ways to separate close-boiling mixtures and has been widely used in the chemical and petroleum industries^16^. The design and optimization of ED is becoming increasingly attractive due to its potential economic advantages. The effectiveness of an ED process relies on the proper choice of solvent^17^. The solvent should be easy to recover and possess a high thermal stability, low toxicity, and high boiling point^18^. The key method for solvent selection is to compare the changes in the degree of relative volatility after adding different solvents. For example, dimethyl sulfoxide is a hydrogen bond breaker that is effective in breaking the azeotropes of tetrahydrofuran-water^18^ and trimethyl borate-methanol^19^, the solvent butyl propionate can greatly enlarge the relative volatility of isobutyl acetate and isobutyl alcohol because the solvent forms a homologous series with isobutyl acetate^20^, and N-methyl-2-pyrrolidone (NMP) was selected as a suitable solvent for n-heptane-isobutanol separation^21^. Residue curve maps (RCMs) can also be used to find suitable solvents for separation processes. Based on an RCM analysis, water was used as the solvent to separate acetone-methanol^22^, and the nontoxic solvent tetraethyleneglycol was used for ethanol-water separation since distillation boundaries did not appear in the RCM^23^.

Optimization and process intensification are the two other factors that should be considered when designing an economic ED process. To reduce the total annual cost (TAC) and achieve further energy saving, methods for process intensification and integration combined with ED have been published in many papers^24–31^ using thermally coupled distillation column^25^, dividing-wall column^26^ and double-effect distillation^31^ techniques. Many simulation platforms, such as Aspen Plus, HYSYS, PRO/II and ChemCAD, have been used for the conceptual design of extractive distillation, and achievements have been made to improve the economics and controllability. Luyben^32^ provided a detailed introduction for creating a steady-state design and optimizing and assessing the controllability of a distillation process using Aspen Plus. Long and Lee^33^ investigated the ED process with a retrofit design using HYSYS to achieve further energy saving and to improve the process capacity. Shirsat *et al*.^34^ rigorously optimized an ED configuration using ChemCAD, and found that the process of ethanol dehydration was more economical using a pre-separator column. Timoshenko *et al*.^25^ investigated several alternative ED processes with and without partially thermally coupled columns for different types of ternary mixtures, and a case study was discussed to select the most energy-saving process using PRO/II software. All of the above studies promoted the development of ED.

**Figure 1:**
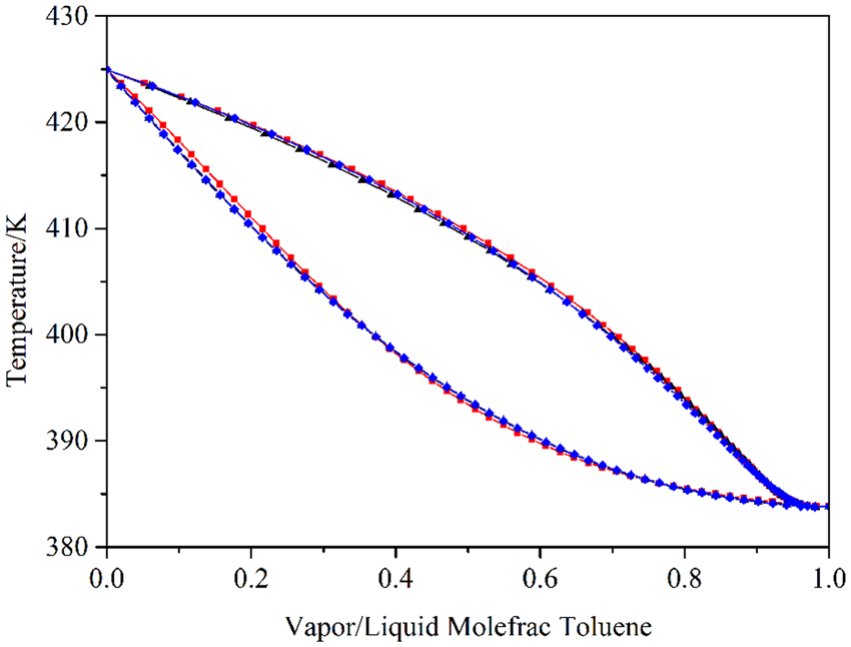
T*-xy* diagram of the Toluene-DMF mixture using the built-in interaction parameters at 1 atm:  NRTL model;  UNIQUAC model; and  Wilson model.

In the separation process, the selection of the thermodynamic model in the simulator software is a primary issue for performing the phase equilibrium calculation^35–38^. Dimethyl formamide (DMF) and NMP are widely used as solvents to design ED processes, and the unusual phase behaviors caused by the different interaction parameters of toluene-DMF, benzene-NMP and cyclohexane-NMP attracted our interest. Mixtures of DMF and nonaromatics, such as cyclohexane and heptane, can form minimum boiling point azeotropes^39^. Luyben^40^ selected DMF as the solvent to design an ED process using the built-in binary interaction parameters of the NRTL model. The calculation with the built-in binary interaction parameters for toluene-DMF in Aspen Plus, however, indicated that the mixture forms a homogeneous minimum boiling azeotrope at atmospheric pressure (Fig. 1). As seen from the experimental data published in Azeotropic Data^41^ at different pressures, the binary system of toluene-DMF does not exhibit azeotropic behavior.

NMP has been chosen as a suitable solvent for separating aromatic and nonaromatic mixtures^42–47^. Methods for solvent selection have demonstrated that NMP efficiently alters the relative volatility of benzene-cyclohexane^42–44^. Vega *et al*.^43^ found that NMP is an efficient solvent for separating benzene-cyclohexane-cyclohexene mixtures using non-steady-state gas chromatography. However, a few studies^40, 48^ have shown that NMP is not an efficient solvent for separating mixtures that contain binary azeotropes of benzene-cyclohexane.

The purpose of this article was to study the effect of various thermodynamic model parameters when designing ED processes for separating mixtures using DMF and NMP solvents. The VLE data that have been reported in the literature^49, 50^ were regressed to obtain the binary interaction parameters in order to demonstrate that the binary system of toluene-DMF does not exhibit azeotropic behavior and that NMP can be used as an appropriate solvent for separating azeotropic mixtures of benzene-cyclohexane during ED. The detailed separation process was carried out using the regressed binary interaction parameters to obtain accurate separation results.

## Methods

### Data regression

Experimental data were obtained using the function from the National Institute of Standards and Technology ThermoData Engine (NIST TDE) in Aspen Plus V8.8. The NRTL, UNIQUAC, and Wilson models were used to correlate the experimental data for this binary system. Area consistency tests were used to check the thermodynamic consistency. Experimental data^49, 50^ for the systems of toluene-DMF, benzene-NMP and cyclohexane-NMP, which passed the thermodynamic area consistency test, were regressed to obtain binary interaction parameters. The root mean square deviation^51, 52^ (RMSD) values were calculated between the experimental and calculated results to select the appropriate thermodynamic model. The non-randomness parameter (α) of the NRTL model was set at different values. Renon and Prausnitz^53^ recommended setting α between 0.2 and 0.5. In many examples in the literature^54, 55^, the values of α_ij_ were smaller than 0.2 to achieve better regression results. A wider interval for α was considered to correlate the experimental data with high accuracy, and the value of α was optimized by minimizing the RMSD.

### Economics

The TAC is the sum of the annualized operating costs and capital costs, and the relevant approximation methods were taken from Douglas^56^. The total capital investments refer to the cost of the column vessels and heat exchangers. The investments required for the valves, reflux drums, pipes, and pumps are usually neglected. The columns and sieve plate parameters are determined using the “Tray Sizing” function. And the first stage is the reflux drum and the last stage is the reboiler. The total heat transfer coefficients of reboilers and condensers are 0.568  kW/(Km^2^) and 0.852 kW/(Km^2^), respectively. Detailed information for calculating the TAC was described in our previous paper^57^.

### Process optimization

**Figure 2:**
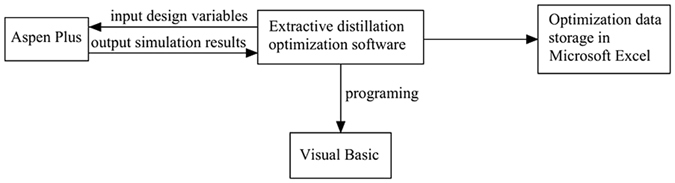
Optimization algorithm interfaces for the Extractive Distillation Optimization Software.

In previous work, we developed software to optimize pressure-swing distillation processes based on simulated annealing algorithms^58^ and sequential iterative optimization procedures^59^. In this study, the influence of the solvent-to-feed ratio was considered during ED, and Extractive Distillation Optimization Software (EDOS)^60^ software was developed to implement program optimization on the basis of a sequential iterative optimization procedure. The EDOS was programmed using a simulation-optimization technique implemented by the Visual Basic interface with Aspen Plus and the optimization algorithm, as shown in Fig. 2. The design variables including the solvent amount, the total number of stages in columns, the feed tray location and the solvent feed stage were optimized to obtain the minimal total annual cost. All the optimization data were saved in Microsoft Excel for further verification and analysis.

## Results and Discussion

### Benzene-cyclohexane-toluene separation using DMF as the solvent

Thermodynamic models such as NRTL, UNIQUAC and Wilson were used to analyze the properties of a ternary mixture with and without DMF. The simulation results showed that DMF and toluene formed a homogeneous minimum boiling azeotrope at atmospheric pressure using different models with the built-in binary interaction parameters of Aspen Plus. The azeotropic composition and temperature were 0.42 mol% DMF and 383.83 K for the NRTL model, 1.54 mol% DMF and 383.78 K for the UNIQUAC model, and 1.87 mol% DMF and 383.72 K for the Wilson model, respectively. For the three models, the azeotropes disappeared at 0.9 atm, 0.7 atm and 0.5 atm, respectively. The azeotropic data were inconsistent with the actual data. Hence, the selection of the thermodynamic model and the determination of the exact binary interaction parameters require more attention to describe the phase behavior of toluene-DMF accurately. Figure 1 shows the *T-xy* diagram for the system of toluene-DMF at 1 atm using the built-in binary interaction parameters of the NRTL, UNIQUAC and Wilson models. However, the experimental data published in Azeotropic Data^41^ indicate that the binary system of toluene-DMF does not exhibit azeotropic behavior under different pressures. Therefore, the built-in binary interaction parameters should be modified.

**Figure 3:**
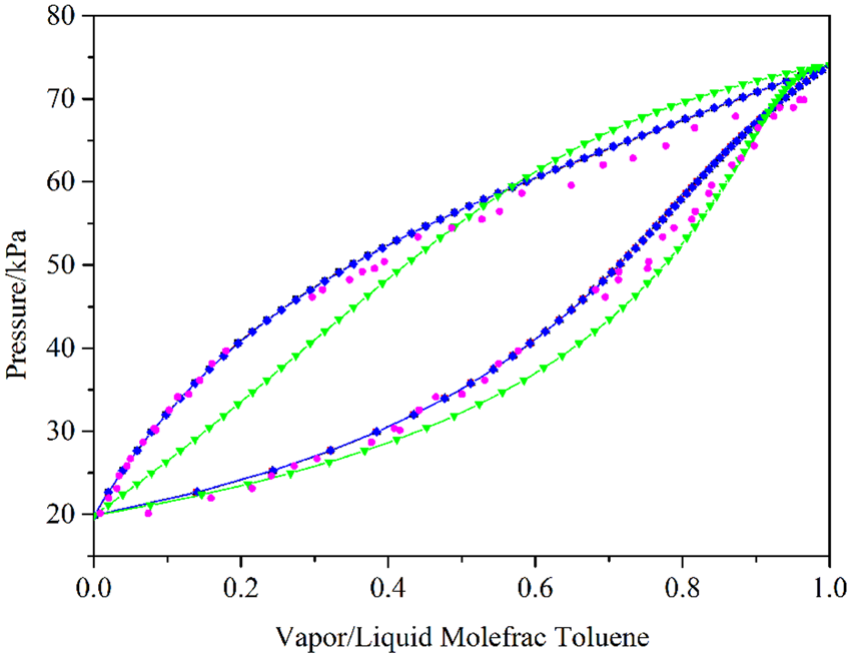
Comparison between the experimental data, correlated results and default results for the system of Toluene-DMF at T = 373.15 K:  experimental data;  correlated results from the NRTL model;  correlated results from the UNIQUAC model;  correlated results from the Wilson model; and  the default results from the NRTL model.

Experimental data for the binary system of toluene-DMF were taken from the work of Yu *et al*.^49^. The obtained binary interaction parameters and RMSD values are shown in Table 1. The average deviations in the pressure and vapor phases using the NRTL model were 0.140 and 0.0119, respectively. The small deviations indicated that the NRTL model with the regressed binary parameters could be used to describe the phase behavior of the toluene-DMF mixture. Figure 2 shows the comparison among the experimental data, correlated results, and default results. The figure shows that the built-in binary interaction parameters produced large deviations in the VLE phase behavior, and the regressed binary interaction parameters described the phase behavior accurately. As shown in Fig. 3, the *xy* curves calculated using the regressed binary interaction parameters did not exhibit azeotropic behavior for the toluene-DMF binary system.

**Table 1:** Correlated model parameters and average absolute deviations for the systems.

Model	a_ij_	a_ji_	b_ij_/K	b_ji_/K	α_ij_	RMSD^a^ (*P*/kPa)	RMSD^a^ (*y*_*i*_)
Toluene (i) + DMF (j)							
NRTL^b^	0	0	332.814	−28.501	0.10	0.140	0.0119
UNIQUAC^c^	−7.297	7.608	2683.662	−2884.069		0.168	0.0118
Wilson^d^	0	0	−125.353	−200.774		0.145	0.0117
Benzene (i) + NMP (j)							
NRTL^b^	0	0	3776.060	−2904.104	0.03	0.026	0.0004
UNIQUAC^c^	−1.510	1.088	666.906	−528.010		0.089	0.0010
Wilson^d^	2.386	−2.442	−1103.648	1121.244		0.076	0.0007
Cyclohexane (i) + NMP (j)							
NRTL^b^	0	0	1085.790	−270.811	0.14	0.027	0.0002
UNIQUAC^c^	9.229	21.900	−3414.744	−7217.892		0.066	0.0001
Wilson^d^	0	0	−181.280	−769.390		0.045	0.0004

${}^{{\rm{a}}}{\rm{RMSD}}={[\frac{1}{{\rm{n}}}\sum _{{\rm{i}}=1}^{{\rm{n}}}{({{\rm{M}}}_{{\rm{i}}}^{\exp }-{{\rm{M}}}_{{\rm{i}}}^{{\rm{cal}}})}^{2}]}^{\frac{1}{2}}$, where n is the total number of data points and M^exp^ and M^cal^ represent the experimental and calculated values, respectively. ^b^NRTL model: τ_ij_ = a_ij_ + b_ij_/T. ^c^UNIQUAC model: τ_ij_ = exp(a_ij_ + b_ij_/T). ^d^Wilson model: In A_ij_ = a_ij_ + b_ij_/T.

**Figure 4:**
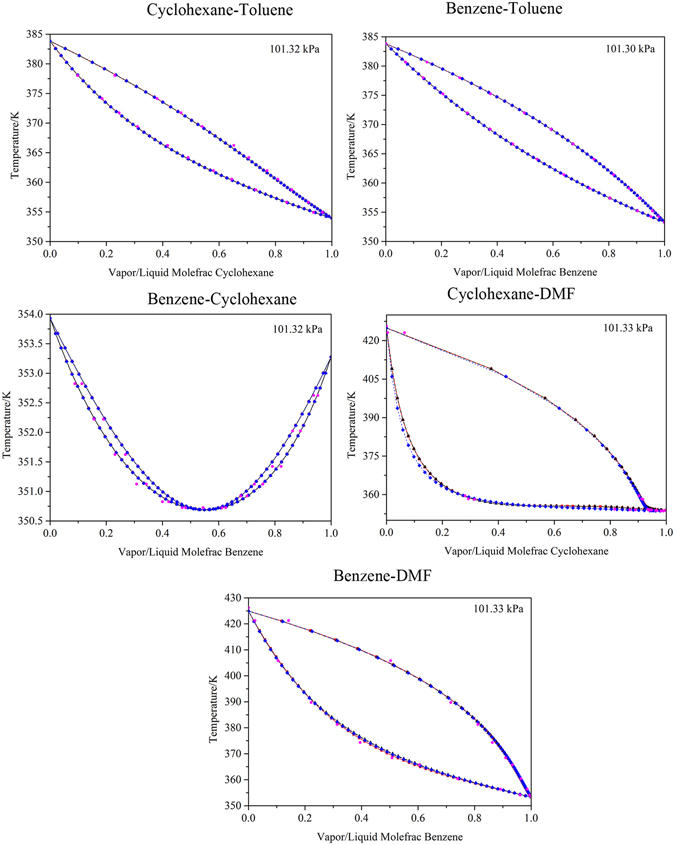
Experimental data and calculated results using the built-in binary interaction parameters for the system of Benzene-Cyclohexane-Toluene-DMF:  experimental data;  NRTL model;  UNIQUAC model; and  Wilson model.

For the binary systems of benzene-cyclohexane, benzene-toluene, cyclohexane-toluene, benzene-DMF and cyclohexane-DMF, the VLE predicted using the built-in binary interaction parameters was compared with the experimental data^41^ to verify the suitability of the thermodynamic model. Figure 4 indicates that the NRTL model with the built-in binary interaction parameters fit the experimental data well. Hence, the parameters can be used to simulate the VLE for benzene-cyclohexane, benzene-toluene, cyclohexane-toluene, benzene-DMF and cyclohexane-DMF systems.

#### Process design

The triple column extractive distillation process using built-in binary interaction parameters (TCEDBBIP) was reported previously for separating the ternary mixture of benzene-cyclohexane-toluene using DMF as the solvent^40^. In this study, triple column extractive distillation using regressed binary interaction parameters (TCEDRBIP) was designed using the same feeding conditions to design the modified separation process. The mixture flow rate was 100 kmol/h with a composition of 30 mol% benzene, 30 mol% cyclohexane and 40 mol% toluene at 323 K. The operating pressure of the first column (C1) was set at 0.6 atm to achieve high-purity cyclohexane because the azeotrope of cyclohexane-DMF disappeared under this pressure. The operating pressure of the second column (C2) was set at 1 atm, and the product of benzene was recovered at the top of the column. The operating pressure of the third column (C3) was set at 0.5 atm to avoid using the medium-pressure stream in the reboiler. The stage pressure drop was set at 0.0068 atm. Three specifications, 0.005 mol% cyclohexane in C1, 99 mol% benzene in C2, and 0.1 mol% toluene in C3, were achieved by varying the reflux ratios of the corresponding columns.

**Figure 5:**
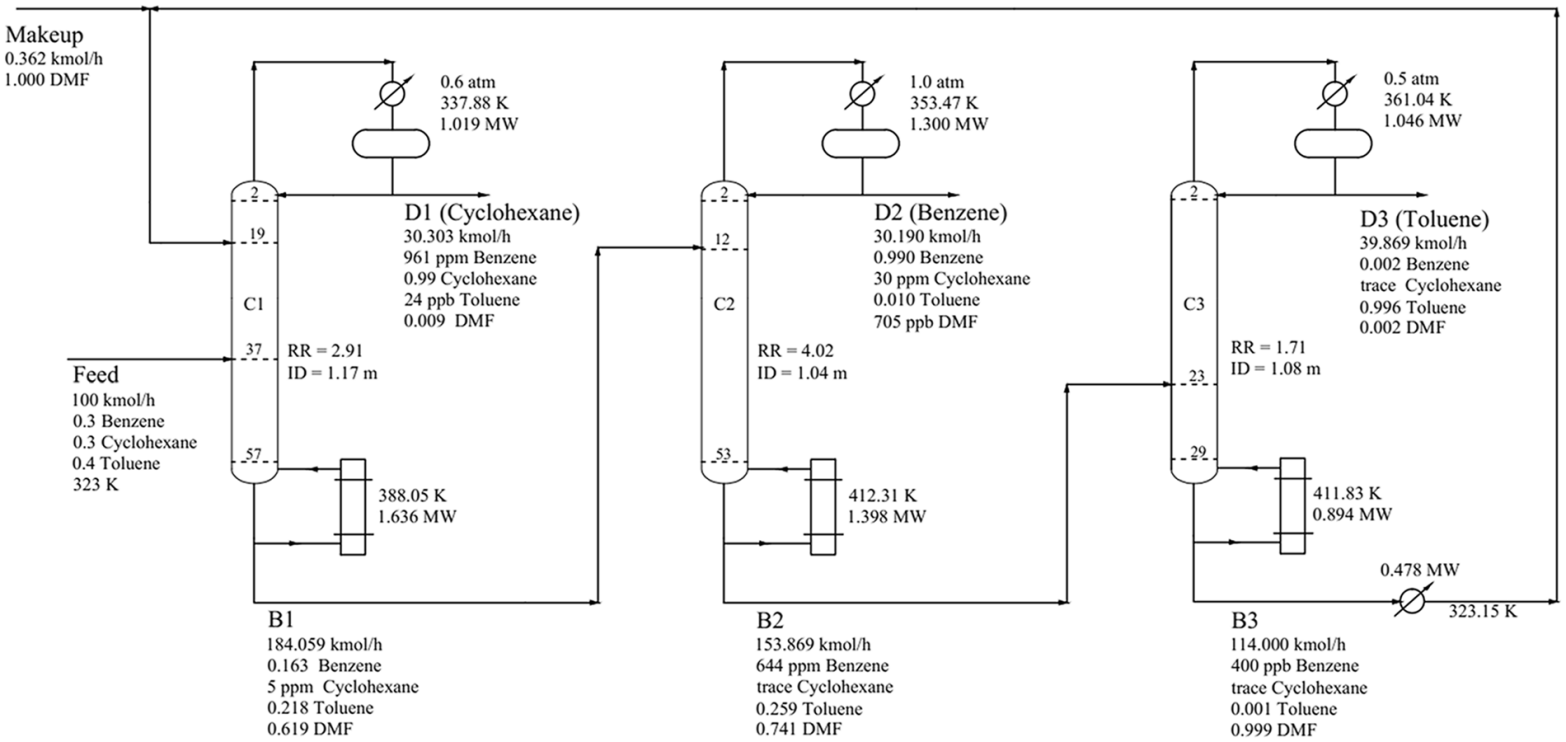
Process flowsheet of TCEDRBIP with details.

**Figure 6:**
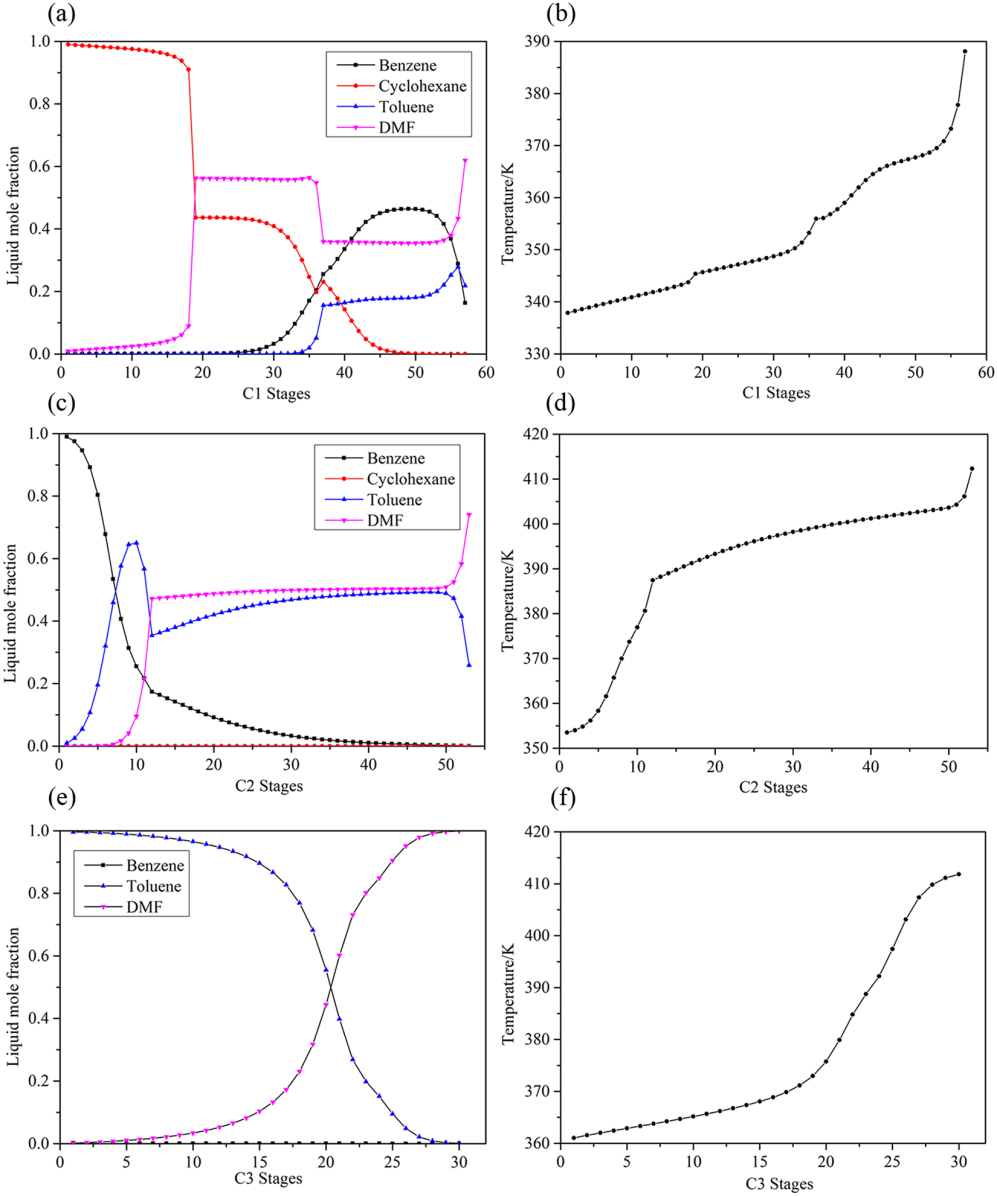
Composition and temperature profiles of TCEDRBIP: (**a**) Composition profiles in C1; (**b**) temperature profile in C1; (**c**) composition profiles in C2; (**d**) temperature profile in C2; (**e**) composition profiles in C3; and (**f**) temperature profile in C3.

The detailed optimization results are shown in Fig. 5 with the operating conditions, heat duties, stream information and equipment sizes. Figure 6 shows the liquid composition and temperature profiles of the three columns. The total reboiler and condenser duties of the columns were 3.928 and 3.365 MW, respectively. The TAC calculated for TCEDRBIP was 1.430 × 10^6^ $/y. The annual operating cost and total capital cost were 9.095 × 10^5^ $/y and 1.562 × 10^6^ $/y, respectively.

#### Process comparison

The data in Table 2 show the optimal parameters of TCEDRBIP and TCEDBBIP. The obtained operating pressure was different in TCEDBBIP^40^. In TCEDBBIP, C1 and C3 were operated at 0.69 atm and 0.12 atm, respectively, due to the existence of azeotropic behavior in the binary system of toluene-DMF. In fact, in TCEDRBIP, azeotropic behavior was not observed for toluene-DMF, and the system of toluene-DMF was effectively separated without requiring very high vacuum.

**Table 2:** Optimal operation parameters and energy consumption of the two processes.

Process	Column	N_T_	N_E_/N_F_	RR	FE kmol/h	Q^reb^ MW	Q^cond^ MW	Pressure atm
TCEDRBIP	Column 1	58	19/37	2.91		1.636	1.019	0.60
	Column 2	54	−/12	4.02	114.00	1.398	1.300	1.00
	Column 3	30	-/23	1.71		0.894	1.046	0.50
TCEDBBIP	Column 1	80	20/40	3.88		1.731	1.251	0.69
	Column 2	41	−/20	5.26	89.74	1.820	1.608	1.08
	Column 3	21	−/15	1.80		0.679	1.137	0.12

TCEDRBIP, which was optimized on the basis of the minimal TAC, required larger amounts of solvent (114.362 kmol/h) than TCEDBBIP, which was designed based on the total energy consumption in the reboilers of the columns. The total energy consumption in the reboilers of TCEDRBIP was 3.928 MW and was lower than that of TCEDBBIP. Stages of 58, 54 and 30 in the three columns of TCEDRBIP needed to reach the specification purity of the products with the lowest TAC. The annual operating cost and the total capital cost of TCEDBBIP were 1.007 × 10^6^ $/y and 1.689 × 10^6^ $/y, respectively. TCEDRBIP incurred 7.13% energy consumption and reduced the TAC by 8.92% compared with TCEDBBIP.

### Benzene-cyclohexane separation with NMP as the solvent

NMP has been used as an efficient solvent for separating the azeotropes of benzene-cyclohexane mixtures. Timoshenko *et al*.^25^ employed NMP to separate the ternary mixture of benzene-cyclohexane-toluene and designed the separation process using PRO/II software with the NRTL thermodynamic model. The ternary mixture contained a binary azeotrope of benzene-cyclohexane, and the important role of adding NMP was to break the azeotrope to improve the relative volatility of benzene and cyclohexane. Timoshenko’s designs were carried out in steady state using the Aspen Plus platform, and the results of the simulation with the built-in binary interaction parameters showed that the separation process was not duplicated using the same optimized parameters. To obtain accurate parameters for the separation process using Aspen Plus, binary interaction parameters of benzene-NMP and cyclohexane-NMP were regressed using VLE data from the literature^50^.

**Figure 7:**
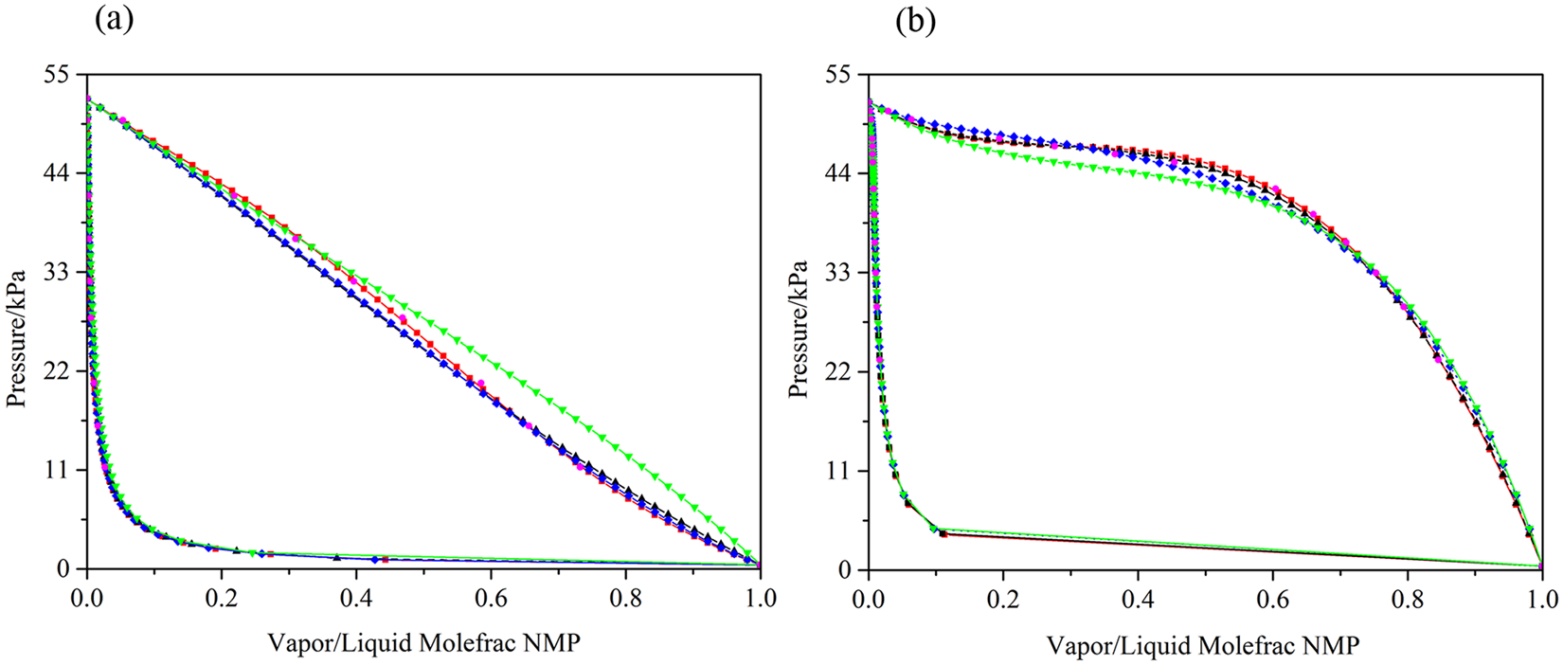
Comparison between the experimental data, correlated results and default results for two systems of (**a**) Benzene-NMP and (**b**) Cyclohexane-NMP at T = 333.25 K:  experimental data;  correlated results from the NRTL model;  correlated results from the UNIQUAC model;  correlated results from the Wilson model; and  the default results from the NRTL model.

Experimental data for the binary systems of benzene-NMP and cyclohexane-NMP, which passed the thermodynamic area consistency test, were taken from the work of Gierycz *et al*.^50^. The binary interaction parameters and RMSD values are shown in Table 1. The average deviations in the pressure using the NRTL model for the two systems were 0.0263 and 0.0271, respectively. The average deviations in the vapor phase using the NRTL model for both systems were 0.000374 and 0.000156, respectively. The small deviations show that the NRTL model can be used to describe the phase behaviors of the two systems. Figure 7a and b show the comparison among the experimental data, correlated results, and default parameter results. As shown in Fig. 7a and b, the vapor behavior curves of the systems calculated with the regressed binary interaction parameters were more satisfied compared with those using the built-in binary interaction parameters in Aspen Plus.

#### Process design

**Figure 8:**
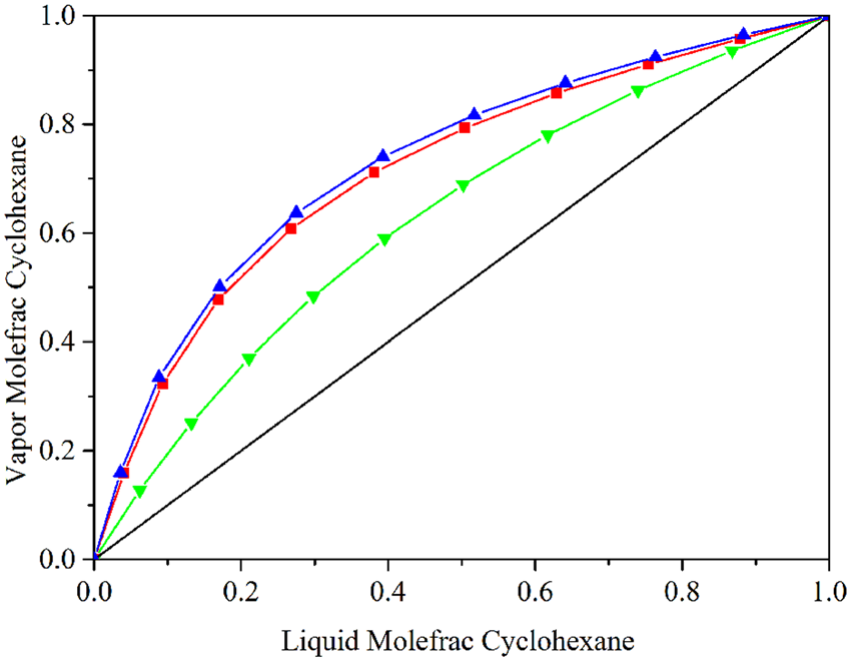
Comparison between the correlated results and default results from the Aspen Plus and PRO/II simulators using the NRTL model for the system of Benzene-Cyclohexane at P = 101.33 kPa:  default parameters from PRO/II;  correlated results from Aspen Plus; and  default results from Aspen Plus.

In this study, double-column extractive distillation using the regressed interaction parameters (DCEDRBIP) and using the built-in binary interaction parameters (DCEDBBIP) were explored to separate benzene-cyclohexane using NMP as a solvent. A Flash 2 model in Aspen Plus was employed to calculate the relative volatility of the benzene and cyclohexane to illustrate the effect of NMP on the azeotrope. The relative volatility values calculated using the regressed and built-in binary interaction parameters were 3.96 and 2.21, respectively. Figure 8 shows the accuracy of the binary interaction parameters for describing the influence of the NMP solvent on the VLE with a solvent to feed mole ratio of 1. The results show that the addition of NMP could enlarge the relative volatility of benzene and cyclohexane. The relative volatility calculated using the built-in binary interaction parameters deviated from the experimental data to a large extent. Furthermore, the relative volatility calculated using the regressed binary interaction parameters was close to the value that was calculated using the built-in binary interaction parameters in the PRO/II software.

**Figure 9:**
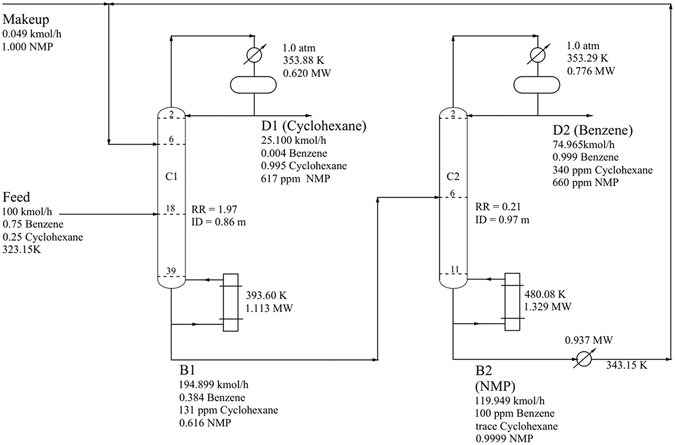
Process flowsheet of DCEDRBIP with details.

**Figure 10:**
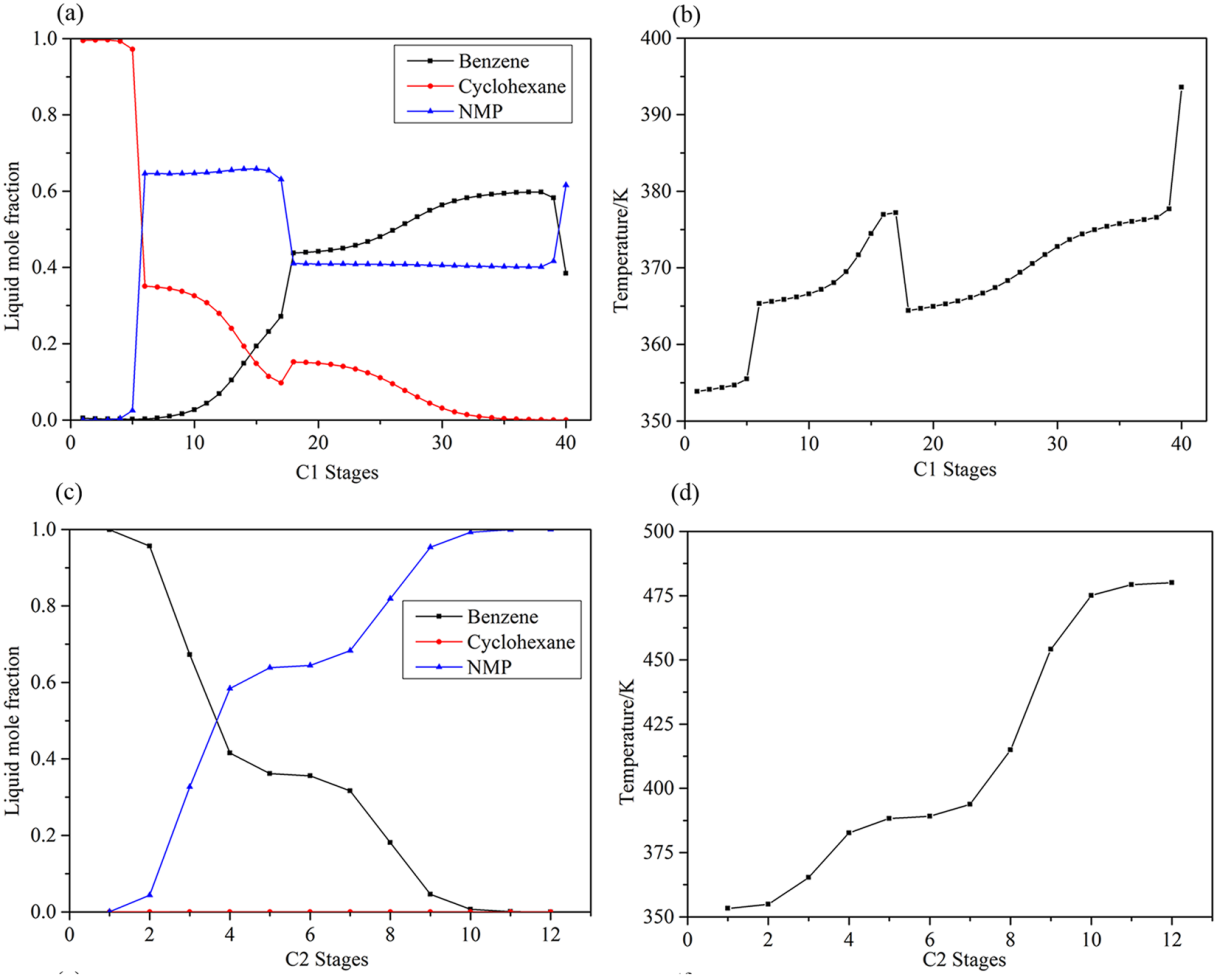
Composition and temperature profiles of TCEDRBIP: (**a**) Composition profiles in C1; (**b**) temperature profile in C1; (**c**) composition profiles in C2; and (**d**) temperature profile in C2.

**Figure 11:**
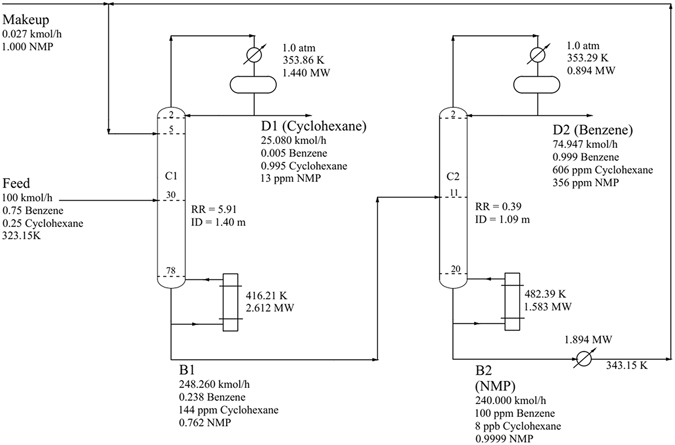
Process flowsheet of DCEDBBIP with details.

**Figure 12:**
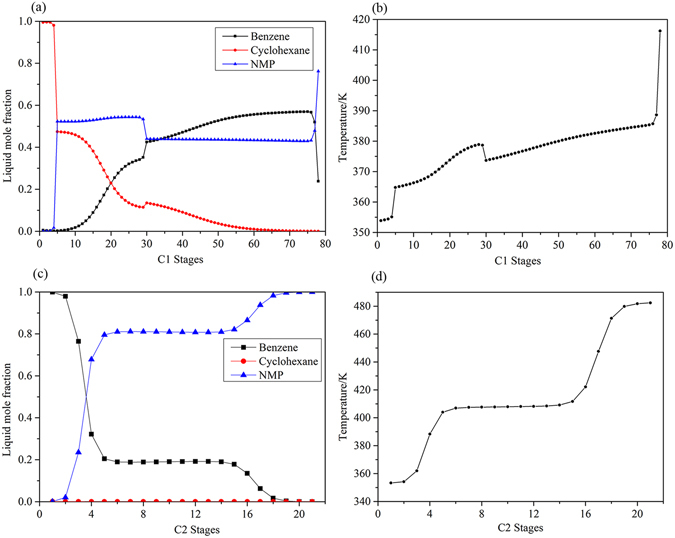
Composition and temperature profiles of DCEDBBIP: (**a**) Composition profiles in C1; (**b**) temperature profile in C1; (**c**) composition profiles in C2; and (**d**) temperature profile in C2.

The mixture flow rate was 100 kmol/h with a composition of 75 mol% benzene and 25 mol% cyclohexane. Both columns were operated at atmospheric pressure, and the tray drop pressure between two adjacent stages was set at 0.0068 atm. The cyclohexane product of the extractive column was specified as 99.5 mol%. For the recovery column, the bottom purity was set at 99.99 mol% NMP. The detailed optimization results for DCEDRBIP are shown in Fig. 9, and the liquid composition and temperature profiles for the DCEDRBIP process with the minimal TAC are shown in Fig. 10. The detailed process of DCEDBBIP is shown in Fig. 11, and Fig. 12 shows the liquid composition and temperature profiles. The reboiler duties of the processes with the regressed and built-in binary interaction parameters were 2.442 and 4.195 MW, respectively, and condenser duties of the two processes were 1.396 and 2.334 MW, respectively. The TAC values calculated for both processes were 7.659 × 10^5^ $/y and 1.402 × 10^6^ $/y, respectively.

#### Process comparison

Although the differences in the relative volatility for the two processes were small, the optimal parameters and energy consumption of the two processes were significantly different, which has a great impact on practical applications. The total number of stages in DCEDRBIP and DCEDBBIP were 52 and 100, respectively. The solvent amount of DCEDRBIP was lower than that of DCEDBBIP. Therefore, the column vessel cost of DCEDRBIP was lower. DCEDRBIP and DCEDBBIP required 2.594 and 4.195 MW of energy in the reboilers, respectively. The difference between the energy consumption of the two processes was large. DCEDRBIP incurred 38.16% energy consumption and reduced the TAC by 45.37% compared with DCEDBBIP.

## Conclusion

Extractive distillation processes using regressed and built-in binary interaction parameters for separating mixtures were investigated. The simulation results using the regressed binary interaction parameters did not indicate azeotropic behavior for the system of toluene-DMF, and the VLE was consistent with the experimental data. The total energy consumption of TCEDRBIP was lower and accounted for 9.70% of the annual operating cost and reduced the TAC by 8.92% compared with TCEDBBIP.

The effect of the amount of NMP on the relative volatility of benzene and cyclohexane was calculated. The relative volatility calculated using the regressed binary interaction parameters was 3.96, which was 1.84 times the value calculated using the built-in binary interaction parameters. The solvent requirements of DCEDRBIP were lower than those of DCEDBBIP. The TAC had large deviations between the two processes. The DCEDRBIP reduced the TAC and energy consumption by 45.37% and 38.16%, respectively.

For some systems, the built-in binary interaction parameters were more accurate for describing the phase behaviors. However, the phase behaviors of some systems described by the built-in binary interaction parameters were obviously different from the experimental data in determining the azeotropic phenomenon or the relative volatility of the components. There were great deviations between the simulated results obtained using the regressed and built-in binary interaction parameters. For systems in which the built-in binary interaction parameters cannot describe the phase behavior accurately, it is more appropriate to use binary interaction parameters regressed by experimental data to design and optimize the separation process. It is important for researchers to carefully study the VLE data and determine the suitable binary interaction parameters when designing distillation processes for azeotrope separation.
